# Association between centre volume and allocation to curative surgery and long-term survival for retroperitoneal sarcoma

**DOI:** 10.1093/bjsopen/zrad059

**Published:** 2023-07-27

**Authors:** Sivesh K Kamarajah, Marco Baia, David N Naumann, Fahad Mahmood, Alessandro Parente, Max Almond, Fabio Tirotta, Samuel J Ford, Fadi Dahdaleh, Anant Desai

**Affiliations:** Midlands Abdominal and Retroperitoneal Sarcoma Unit, Queen Elizabeth Hospital, Birmingham, UK; Academic Department of Surgery, Institute of Applied Health Research, University of Birmingham, Birmingham, UK; Midlands Abdominal and Retroperitoneal Sarcoma Unit, Queen Elizabeth Hospital, Birmingham, UK; Midlands Abdominal and Retroperitoneal Sarcoma Unit, Queen Elizabeth Hospital, Birmingham, UK; Midlands Abdominal and Retroperitoneal Sarcoma Unit, Queen Elizabeth Hospital, Birmingham, UK; Midlands Abdominal and Retroperitoneal Sarcoma Unit, Queen Elizabeth Hospital, Birmingham, UK; Midlands Abdominal and Retroperitoneal Sarcoma Unit, Queen Elizabeth Hospital, Birmingham, UK; Midlands Abdominal and Retroperitoneal Sarcoma Unit, Queen Elizabeth Hospital, Birmingham, UK; Midlands Abdominal and Retroperitoneal Sarcoma Unit, Queen Elizabeth Hospital, Birmingham, UK; Edward-Elmhurst Health Hospital, Chicago, Illinois, USA; Midlands Abdominal and Retroperitoneal Sarcoma Unit, Queen Elizabeth Hospital, Birmingham, UK

## Introduction

Retroperitoneal sarcomas (RPS) are rare tumours arising from mesenchymal cells in the retroperitoneum, encompassing a wide range of different histologies. According to the latest consensus statements from the Transatlantic Australasian RPS Working Group (TARPSWG), curative surgical resection is the cornerstone of treatment and should be performed in specialist centres by expert sarcoma surgeons to ensure appropriate treatment within a multidisciplinary setting^1^. Moreover, including patients in clinical trials and prospective data collection, routinely performed in high-volume centres, drives the advance of knowledge in this field as demonstrated by the collaborative efforts of the TARPSWG^2,3^. Surgery is the best curative treatment for RPS. Oncological treatments such as preoperative radiotherapy do not seem to improve survival for all RPS, and the role of neoadjuvant chemotherapy is currently being investigated^4,5^. As a direct consequence of this, surgery for primary RPS is expected to be the treatment of choice in referral centres, with only a few inoperable cases treated with best supportive care^6^. Survival of these patients who are not operated on is dismal.

Using the National Cancer Database (NCDB) from the USA, the aim of this study was to investigate whether a higher volume of patients and referrals was associated with higher allocation to surgery and better survival.

## Methods

The NCDB, a joint project of the Commission on Cancer of the American College of Surgeons and the American Cancer Society, was used to identify patients diagnosed with a non-metastatic RPS according to the International Classification of Diseases for Oncology, Third Edition (ICD-O-3) who received either surgery or no surgery between 2004 and 2016. Details of data collected and statistical analysis are available in *Appendix S1*.

## Results

### Baseline characteristics

This study included 11 254 patients with a diagnosis of RPS in the study interval, during which 64.1 per cent underwent surgery. Baseline characteristics are summarized in *Table S1*. The most frequent age category in the study population was under 55 years (26.9 per cent); 49.1 per cent were male. Patients were mainly of white race (83.9 per cent) and with no co-morbidities (76.7 per cent with Charlson/Deyo co-morbidity score = 0). The majority of patients were insured either with a private provider (43.3 per cent) or Medicare (42.7 per cent), and 6.8 per cent were uninsured. Zip code-level education status was mainly in the 7–12.9 per cent range (30.4 per cent) and the household income was greater than or equal to $63 000 in 39.8 per cent of cases. Considering the tumour characteristics, the most common histology was liposarcoma (53.2 per cent) and the most frequent stage at presentation was cT4 (38.0 per cent).

### Patients allocated to surgery

**Fig. 1:**
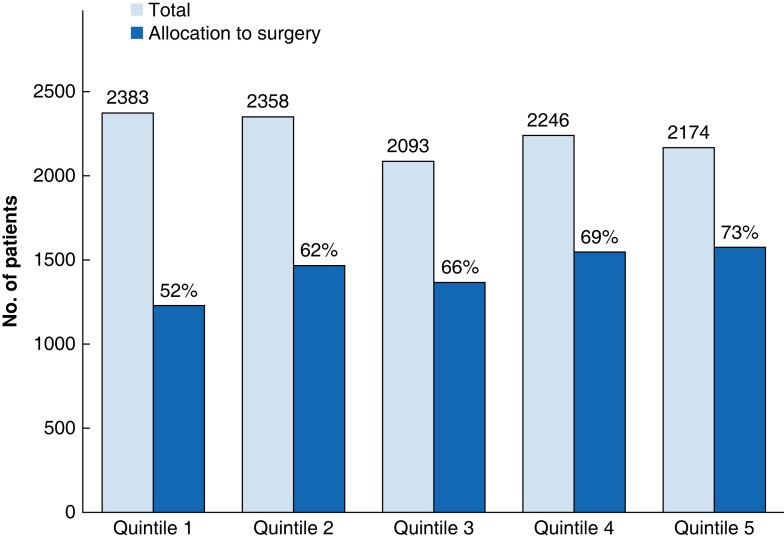
Histogram of centre-volume quintiles and allocation to surgery for patients with retroperitoneal sarcoma

Patients receiving surgery for RPS were more likely to be from high-volume centres (that is quintile 5: 73 per cent) compared with low-volume centres (that is quintile 1: 52 per cent; *P* < 0.001, *Fig. 1*) and academic centres compared with community centres (58 *versus* 26 per cent; *P* < 0.001). Patients less likely to have surgery were non-white, male, and greater than 75 years old, with advanced co-morbidities (Charlson/Deyo co-morbidity score greater than 1), with no insurance or Medicaid, with low household income and education level, and from rural and urban areas of residence. Tumours less likely to be allocated to surgery had a histology other than liposarcoma and were small in size (that is AJCC less than cT3) (*Table S1*).

### Allocation to surgery by centre volume

Stratified analyses were performed to understand patient- and tumour-related factors associated with allocation to surgery. On multivariable logistic regression, centre volume (OR 1.85, 95 per cent c.i. 1.55 to 2.20; *P* < 0.001), female sex (OR 1.26, 95 per cent c.i. 1.16 to 1.38; *P* < 0.001), and greater tumour size (cT4: OR 2.22, 95 per cent c.i. 1.98 to 2.49; *P* < 0.001) were all relevant factors in the allocation of patients to surgery. Private insurance (OR 1.15, 95 per cent c.i. 1.01 to 1.31; *P* = 0.038) and high education level were also associated with allocation to surgery. Conversely, age greater than or equal to 75 years at the time of diagnosis (OR 0.68, 95 per cent c.i. 0.57 to 0.82; *P* < 0.001), non-white race (OR 0.88, 95 per cent c.i. 0.78 to 0.99; *P* = 0.029), and having a diagnosis other than liposarcoma (OR 0.59, 95 per cent c.i. 0.53 to 0.66; *P* < 0.001) meant that patients were less likely to receive surgical treatment (*Table S2*).

### Long-term survival

**Fig. 2:**
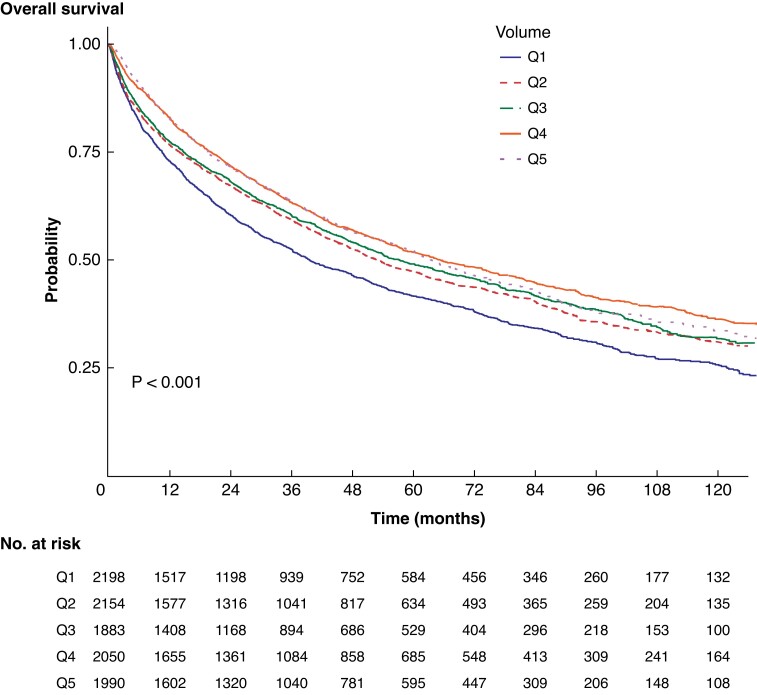
Overall survival of patients with retroperitoneal sarcoma divided in terms of centre-volume quintiles Q, quintile.

High-volume centres were associated with improvement in long-term survival (*Fig. 2*). Multivariate Cox regression analysis showed that factors associated with better 5-year survival in patients with RPS were female sex (OR 0.80, 95 per cent c.i. 0.75 to 0.84; *P* < 0.001), rural residence (OR 0.81, 95 per cent c.i. 0.72 to 0.92; *P* < 0.001), and allocation to surgery (OR 0.60, 95 per cent c.i. 0.57 to 0.64; *P* < 0.001) (*Table S3*). Factors associated with adverse survival were higher age at diagnosis (age greater than or equal to 85 years: OR 3.00, 95 per cent c.i. 2.59 to 3.48; *P* < 0.001), Charlson/Deyo co-morbidity score >1 (OR 1.73, 95 per cent c.i. 1.45 to 2.08; *P* < 0.001), education level less than 7 per cent (OR 1.20, 95 per cent c.i. 1.09 to 1.32; *P* < 0.001), leiomyosarcoma (OR 1.60, 95 per cent c.i. 1.49 to 1.71; *P* < 0.001), and cT4 tumours (OR 1.26, 95 per cent c.i. 1.17 to 1.36; *P* < 0.001).

## Discussion

The main finding from the present study utilizing the NCDB including more than 11 000 patients with non-metastatic RPS is that high institutional cancer volume is associated with significantly higher rates of allocation to surgery and long-term survival. Although a greater overall number of patients were treated in low-volume centres, those referred to high-volume centres experienced a higher rate of allocation to surgical treatment and better overall survival, confirmed in multivariate analysis. The volume–outcome relationship in the context of resection volume and short-term outcome is well described more recently within complex cancer surgery^7,8^. However, there is a lack of data characterizing the relationship between RPS centre volume by number of cancers diagnosed or rates of curative resection and long-term cancer survival to date.

While specific aspects of clinical practice within high-volume centres that might be associated with improved rates of curative resection and neoadjuvant therapy remain uncertain, institutional factors (that is volume and facility type) possibly reflect an environment of focused high-quality oncological care that may influence survival more directly. Two possible explanations have been previously proposed. First, ‘selective referral’ of patients to hospitals that already have improved outcomes would result in a higher volume of patients with survival benefits in these settings^9,10^. Second, continuous referral of patients to experienced centres leads to a constant improvement in the multidisciplinary management of patients across the whole treatment pathway^9^. Higher-volume cancer centres have a restless drive to improve the processes of care along defined multidisciplinary protocols, while increasing the technical experience of surgeons in performing complex cancer surgery and optimizing management of postoperative care including complications. A further consideration is also the direct access to clinical trials in these centres.

High-volume referral centres have some advantages that may lead to improved care for patients, as observed in the current study. First, the increased expertise and continuous training of each surgeon is constantly developed by performing complex surgeries according to recognized oncological principles. Second, prompt availability of a cohort of allied healthcare providers and institutional support facilitates adequate postoperative management, with timely diagnosis and treatment of complications leading to reduced perioperative morbidity and mortality. Last, an organized network involving the referring hospital can coordinate adequate surveillance with punctual diagnosis and treatment of recurrence^11–14^. Multimodality cancer staging, appropriate use of combined oncological strategies (including neoadjuvant therapy), and improved surgical techniques all play a role in the improved benefit observed^10,15^.

High-institutional-cancer-volume centres are independently associated with both increased allocation to surgical therapy and better long-term survival for patients with RPS. As hospitals affiliate in response to broader financial and political pressures, in both the USA and Europe, these systems may present opportunities to improve the quality of care for these patients.

## Supplementary Material

zrad059_Supplementary_Data

## Data Availability

Data are available on request.
