# Supplementary material for: Association between centre volume and allocation to curative surgery and long-term survival for retroperitoneal sarcoma
Source: BJS Open. 2023 Jul 27;7(4):zrad059. doi: 10.1093/bjsopen/zrad059 (PMC10373904; doi:10.1093/bjsopen/zrad059)
Supplement: zrad059_Supplementary_Data [file zrad059_supplementary_data.docx]

**Association between Centre Volume and Allocation to Curative Surgery and Long-Term Survival for Retroperitoneal Sarcoma**

Sivesh K. Kamarajah*^1, 2^, Marco Baia*^1^, David N Naumann^1^, Fahad Mahmood^1^, Alessandro Parente^1^, Max Almond^1^, Fabio Tirotta^1^, Samuel J Ford^1^, Fadi Dahdaleh^3^, Anant Desai^1^

1. Midlands Abdominal and Retroperitoneal Sarcoma Unit, Queen Elizabeth Hospital, Birmingham, UK
2. Institute of Cancer and Genomic Sciences, University of Birmingham, Birmingham, UK
3. Edward-Elmhurst Health Hospital, Chicago, IL, USA

** both authors contributed equally*

**Corresponding author**

Mr Anant Desai MD FRCS

Midlands Abdominal and Retroperitoneal Sarcoma Unit,

Queen Elizabeth Hospital,

Birmingham, B15 2TH

United Kingdom

**Email:** [Anant.Desai@uhb.nhs.uk](mailto:Anant.Desai@uhb.nhs.uk)

**Supplementary Materials - Index**

| **Supplementary Appendixes** |  |
| --- | --- |
| Appendix S1 | *Page 2* |
| **Supplementary Figures and Tables** |  |
| Table S1 Baseline characteristics of patients with retroperitoneal sarcoma stratified by receipt of surgery | *Page 5* |
| Table S2 Univariable and multivariable logistic regression on factors associated with allocation to surgery in patients with retroperitoneal sarcoma | *Page 7* |
| Table S3. Univariable and multivariable Cox regression on long-term overall survival in patients with sarcoma tumors | *Page 9* |
|  |  |

**Appendix S1**

*Data Source*

A large 13-year observational database study was undertaken using the National Cancer Database (NCDB), which is a joint project of the Commission on Cancer (CoC) of the American College of Surgeons and the American Cancer Society^19,20^. The NCDB gathers information from approximately 1,500 CoC-accredited hospitals and includes more than 70% of all newly diagnosed malignancies in the United States of America. It contains specific details about patient demographics (age, sex, race, insurance status), facility type and location, tumor characteristics (size, grade, stage, histology), treatment course (type of surgery, receipt of chemotherapy, and radiation therapy), and outcomes (resection margins, lymph node status, length of stay, short and long-term mortality).

*Study Population*

*Inclusion criteria*

All adult patients diagnosed with a clinically staged, non-metastatic RPS according to the International Classification of Disease for Oncology, Third Edition (ICD-O-3) who received either surgery or no surgery between 2004 and 2016 in the de-identified NCDB were included.

*Exclusion criteria*

The exclusion criteria were: (i) Any histology other than soft tissue sarcoma such as adenocarcinoma, squamous cell carcinoma, mucinous tumors, neuroendocrine tumors, gastrointestinal stromal tumours (GIST) and other (ii) other concurrent cancer diagnoses; and (iii) metastatic stage at presentation.

*Study Definitions*

The following patient-level characteristics as provided by NCDB were analyzed: age (18 - 35, 36 - 50, 51 - 65, 66 - 80, ≥81 years old), race (white, other), Charlson/Deyo comorbidity score^21^, year of diagnosis, insurance status (Medicare, Medicaid, Private Insurance, Uninsured), zip code-level education status (<7%, 7% - 12.9%, 13% - 20.9%, ≥21%), zip code-level median household income (<$48,000, $48,000 - $62,999, ≥$63,000), and area of residence (urban versus rural). The zip-code level education status represents the proportion of adults in the patient's zip code who did not graduate from high school and is categorized as equally proportioned quartiles among all US zip codes. Hospital-level characteristics were analyzed as follows: facility type (academic, community, other), facility location (Midwest, Northeast, South, West), and hospital distance (<12.5, 12.5 - 49.9, ≥50.0 miles). Finally, the NCBD provides the following clinicopathologic characteristics: clinical T (T0-1, T2, T3, T4, Tx) and N status (N0, N+, Nx), tumor grade/differentiation (well/moderate, poor/anaplastic, unknown), margin status (positive, negative, unknown) and lymphovascular invasion (absent, present, unknown) according to the AJCC 7^th^ version^14^. Quintiles were defined into five groups as follows: Quintile 1 (1 - 7 cases/year), Quintile 2 (8 - 16 cases/year), Quintile 3 (17 - 34 cases/year), Quintile 4 (35 - 91 cases/year) and Quintile 5 (≥92 cases/year).

*Outcomes*

The outcomes of interest were allocation to surgery (defined as undergoing surgery with curative intent during their hospital admission) and long-term survival (both at 5 and 10 years from the date of surgery).

*Data Analysis*

Categorical variables were compared using the chi-squared test. Non-normally distributed continuous data were analyzed using the Mann-Whitney U test. Annual center volume quintiles for all patients were divided equally into five groups and these were reported for each quintile. Survival was estimated using Kaplan-Meier survival curves and compared using the log-rank test. Multivariable analyses were undertaken using Cox proportional hazards models and logistic regression models. Sensitivity analyses were performed for patients undergoing surgery only. A *p*-value of <0.05 was considered statistically significant.

Data analysis was performed using R Foundation Statistical software (R 3.2.2) with TableOne, ggplot2, Hmisc, Matchit and survival packages (R Foundation for Statistical Computing, Vienna, Austria) as previously reported^23^.

**Supplementary Figures and Tables**

| Table S1 Baseline characteristics of patients with retroperitoneal sarcoma stratified by receipt of surgery | | | | | |
| --- | --- | --- | --- | --- | --- |
|  |  | No | Yes | Total | p |
| Center volume | Quintile 1 | 1151 (28.5) | 1232 (17.1) | 2383 (21.2) | <0.001 |
|  | Quintile 2 | 886 (21.9) | 1472 (20.4) | 2358 (21.0) |  |
|  | Quintile 3 | 721 (17.8) | 1372 (19.0) | 2093 (18.6) |  |
|  | Quintile 4 | 694 (17.2) | 1552 (21.5) | 2246 (20.0) |  |
|  | Quintile 5 | 593 (14.7) | 1581 (21.9) | 2174 (19.3) |  |
| Facility Type | Community | 1477 (36.5) | 1897 (26.3) | 3374 (30.0) | <0.001 |
|  | Integrated | 669 (16.5) | 1147 (15.9) | 1816 (16.1) |  |
|  | Academic | 1899 (46.9) | 4165 (57.8) | 6064 (53.9) |  |
| Facility Location | Northeast | 806 (19.9) | 1538 (21.3) | 2344 (20.8) | 0.001 |
|  | Midwest | 875 (21.6) | 1701 (23.6) | 2576 (22.9) |  |
|  | South | 1497 (37.0) | 2399 (33.3) | 3896 (34.6) |  |
|  | West | 867 (21.4) | 1571 (21.8) | 2438 (21.7) |  |
| Hospital Distance | <12.5 miles | 2164 (53.5) | 3256 (45.2) | 5420 (48.2) | <0.001 |
|  | 12.5-49.9 miles | 1169 (28.9) | 2236 (31.0) | 3405 (30.3) |  |
|  | >/=50 miles | 712 (17.6) | 1717 (23.8) | 2429 (21.6) |  |
| Year of Diagnosis | 2004-2005 | 460 (11.4) | 746 (10.3) | 1206 (10.7) | 0.207 |
|  | 2006-2007 | 468 (11.6) | 907 (12.6) | 1375 (12.2) |  |
|  | 2008-2009 | 539 (13.3) | 987 (13.7) | 1526 (13.6) |  |
|  | 2010-2011 | 587 (14.5) | 1059 (14.7) | 1646 (14.6) |  |
|  | 2012-2013 | 624 (15.4) | 1095 (15.2) | 1719 (15.3) |  |
|  | 2014-2015 | 358 (8.9) | 569 (7.9) | 927 (8.2) |  |
|  | 2016-2017 | 1009 (24.9) | 1846 (25.6) | 2855 (25.4) |  |
| Age at diagnosis | <55 | 982 (24.3) | 2045 (28.4) | 3027 (26.9) | <0.001 |
|  | 55-59 | 426 (10.5) | 960 (13.3) | 1386 (12.3) |  |
|  | 60-64 | 526 (13.0) | 1076 (14.9) | 1602 (14.2) |  |
|  | 65-69 | 542 (13.4) | 1066 (14.8) | 1608 (14.3) |  |
|  | 70-74 | 505 (12.5) | 860 (11.9) | 1365 (12.1) |  |
|  | 75-79 | 453 (11.2) | 637 (8.8) | 1090 (9.7) |  |
|  | 80-84 | 346 (8.6) | 411 (5.7) | 757 (6.7) |  |
|  | 85+ | 265 (6.6) | 154 (2.1) | 419 (3.7) |  |
| Sex | Male | 2063 (51.0) | 3459 (48.0) | 5522 (49.1) | 0.002 |
|  | Female | 1982 (49.0) | 3750 (52.0) | 5732 (50.9) |  |
| Race | White | 3324 (82.2) | 6114 (84.8) | 9438 (83.9) | <0.001 |
|  | Other | 721 (17.8) | 1095 (15.2) | 1816 (16.1) |  |
| CDCC | 0 | 3067 (75.8) | 5561 (77.1) | 8628 (76.7) | 0.133 |
|  | 1-2 | 883 (21.8) | 1511 (21.0) | 2394 (21.3) |  |
|  | 2+ | 95 (2.3) | 137 (1.9) | 232 (2.1) |  |
| Insurance Status | Medicare | 1926 (47.6) | 2882 (40.0) | 4808 (42.7) | <0.001 |
|  | Medicaid | 256 (6.3) | 402 (5.6) | 658 (5.8) |  |
|  | Private | 1504 (37.2) | 3371 (46.8) | 4875 (43.3) |  |
|  | Not Insured / Other | 299 (7.4) | 461 (6.4) | 760 (6.8) |  |
|  | (Missing) | 60 (1.5) | 93 (1.3) | 153 (1.4) |  |
| Education level | >21% | 903 (22.3) | 1659 (23.0) | 2562 (22.8) | 0.002 |
|  | 13%-20.9% | 978 (24.2) | 1547 (21.5) | 2525 (22.4) |  |
|  | 7%-12.9% | 1236 (30.6) | 2185 (30.3) | 3421 (30.4) |  |
|  | <7% | 928 (22.9) | 1818 (25.2) | 2746 (24.4) |  |
| Medical Income | </=$47,999 | 1534 (37.9) | 2481 (34.4) | 4015 (35.7) | <0.001 |
|  | $48,000-$62,999 | 1017 (25.1) | 1745 (24.2) | 2762 (24.5) |  |
|  | $63,000 + | 1494 (36.9) | 2983 (41.4) | 4477 (39.8) |  |
| Residence | Metro | 3309 (81.8) | 5724 (79.4) | 9033 (80.3) | <0.001 |
|  | Urban | 516 (12.8) | 921 (12.8) | 1437 (12.8) |  |
|  | Rural | 220 (5.4) | 564 (7.8) | 784 (7.0) |  |
| Tumor Histology | Liposarcoma | 1578 (39.0) | 4412 (61.2) | 5990 (53.2) | <0.001 |
|  | Leiomyosarcoma | 1240 (30.7) | 1834 (25.4) | 3074 (27.3) |  |
|  | Other Histology | 253 (6.3) | 341 (4.7) | 594 (5.3) |  |
|  | Sarcoma NOS | 974 (24.1) | 622 (8.6) | 1596 (14.2) |  |
| AJCC Clinical T Stage | cT1 | 1454 (35.9) | 1823 (25.3) | 3277 (29.1) | <0.001 |
|  | cT2 | 807 (20.0) | 1075 (14.9) | 1882 (16.7) |  |
|  | cT3 | 685 (16.9) | 1133 (15.7) | 1818 (16.2) |  |
|  | cT4 | 1094 (27.0) | 3178 (44.1) | 4272 (38.0) |  |
|  | (Missing) | 5 (0.1) | 0 (0.0) | 5 (0.0) |  |
| AJCC Clinical N Stage | cN0 | 2526 (62.4) | 5040 (69.9) | 7566 (67.2) | <0.001 |
|  | cN1 | 313 (7.7) | 146 (2.0) | 459 (4.1) |  |
|  | cNx | 1206 (29.8) | 2023 (28.1) | 3229 (28.7) |  |

| **Table S2** Univariable and multivariable logistic regression on factors associated with allocation to surgery in patients with retroperitoneal sarcoma | | | |
| --- | --- | --- | --- |
|  |  | OR (univariable) | OR (multivariable) |
| Center volume | Quintile 1 | - | - |
|  | Quintile 2 | 1.55 (1.38-1.74, p<0.001) | 1.42 (1.25-1.61, p<0.001) |
|  | Quintile 3 | 1.78 (1.58-2.01, p<0.001) | 1.55 (1.34-1.80, p<0.001) |
|  | Quintile 4 | 2.09 (1.85-2.36, p<0.001) | 1.55 (1.32-1.83, p<0.001) |
|  | Quintile 5 | 2.49 (2.20-2.82, p<0.001) | 1.85 (1.55-2.20, p<0.001) |
| Facility Type | Community | - | - |
|  | Integrated | 1.33 (1.19-1.50, p<0.001) | 1.12 (0.98-1.29, p=0.097) |
|  | Academic | 1.71 (1.57-1.86, p<0.001) | 1.05 (0.92-1.20, p=0.440) |
| Facility Location | Northeast | - | - |
|  | Midwest | 1.02 (0.91-1.15, p=0.757) | 1.08 (0.95-1.23, p=0.252) |
|  | South | 0.84 (0.75-0.93, p=0.001) | 0.93 (0.82-1.05, p=0.219) |
|  | West | 0.95 (0.84-1.07, p=0.394) | 1.00 (0.87-1.14, p=0.965) |
| Hospital Distance | <12.5 miles | - | - |
|  | 12.5-49.9 miles | 1.27 (1.16-1.39, p<0.001) | 1.15 (1.04-1.27, p=0.008) |
|  | >/=50 miles | 1.60 (1.45-1.78, p<0.001) | 1.27 (1.10-1.46, p=0.001) |
| Year of Diagnosis | 2004-2005 | - | - |
|  | 2006-2007 | 1.20 (1.02-1.40, p=0.030) | 1.14 (0.96-1.35, p=0.145) |
|  | 2008-2009 | 1.13 (0.97-1.32, p=0.128) | 1.00 (0.84-1.19, p=0.999) |
|  | 2010-2011 | 1.11 (0.95-1.30, p=0.175) | 0.97 (0.81-1.15, p=0.702) |
|  | 2012-2013 | 1.08 (0.93-1.26, p=0.310) | 0.88 (0.74-1.05, p=0.150) |
|  | 2014-2015 | 0.98 (0.82-1.17, p=0.822) | 0.78 (0.64-0.95, p=0.015) |
|  | 2016-2017 | 1.13 (0.98-1.30, p=0.090) | 1.21 (1.03-1.44, p=0.023) |
| Age at diagnosis | <55 | - | - |
|  | 55-59 | 1.08 (0.94-1.24, p=0.259) | 1.01 (0.87-1.17, p=0.945) |
|  | 60-64 | 0.98 (0.86-1.12, p=0.786) | 0.98 (0.85-1.13, p=0.771) |
|  | 65-69 | 0.94 (0.83-1.07, p=0.383) | 0.92 (0.78-1.09, p=0.325) |
|  | 70-74 | 0.82 (0.72-0.93, p=0.003) | 0.86 (0.72-1.02, p=0.088) |
|  | 75-79 | 0.68 (0.59-0.78, p<0.001) | 0.68 (0.57-0.82, p<0.001) |
|  | 80-84 | 0.57 (0.49-0.67, p<0.001) | 0.61 (0.50-0.75, p<0.001) |
|  | 85+ | 0.28 (0.23-0.34, p<0.001) | 0.30 (0.23-0.39, p<0.001) |
| Sex | Male | - | - |
|  | Female | 1.13 (1.04-1.22, p=0.002) | 1.26 (1.16-1.38, p<0.001) |
| Race | White | - | - |
|  | Other | 0.83 (0.74-0.92, p<0.001) | 0.88 (0.78-0.99, p=0.029) |
| CDCC | 0 | - | - |
|  | 1-2 | 0.94 (0.86-1.04, p=0.228) | 1.01 (0.91-1.12, p=0.900) |
|  | 2+ | 0.80 (0.61-1.04, p=0.091) | 0.95 (0.71-1.27, p=0.717) |
| Insurance Status | Medicare | - | - |
|  | Medicaid | 1.05 (0.89-1.24, p=0.571) | 0.92 (0.75-1.13, p=0.436) |
|  | Private | 1.50 (1.38-1.63, p<0.001) | 1.15 (1.01-1.31, p=0.038) |
|  | Not Insured / Other | 1.03 (0.88-1.21, p=0.708) | 0.79 (0.65-0.96, p=0.016) |
| Education level | >21% | - | - |
|  | 13%-20.9% | 0.86 (0.77-0.96, p=0.010) | 0.86 (0.76-0.98, p=0.019) |
|  | 7%-12.9% | 0.96 (0.86-1.07, p=0.480) | 0.90 (0.79-1.02, p=0.104) |
|  | <7% | 1.07 (0.95-1.19, p=0.266) | 0.94 (0.81-1.07, p=0.345) |
| Medical Income | </=$47,999 | - | - |
|  | $48,000-$62,999 | 1.06 (0.96-1.17, p=0.247) | 1.04 (0.92-1.17, p=0.537) |
|  | $63,000 + | 1.23 (1.13-1.35, p<0.001) | 1.12 (0.99-1.26, p=0.077) |
| Residence | Metro | - | - |
|  | Urban | 1.03 (0.92-1.16, p=0.596) | 0.99 (0.86-1.14, p=0.838) |
|  | Rural | 1.48 (1.26-1.74, p<0.001) | 1.22 (1.02-1.47, p=0.032) |
| Tumor Histology | Liposarcoma | - | - |
|  | Leiomyosarcoma | 0.53 (0.48-0.58, p<0.001) | 0.59 (0.53-0.66, p<0.001) |
|  | Other Histology | 0.48 (0.41-0.57, p<0.001) | 0.54 (0.45-0.65, p<0.001) |
|  | Sarcoma NOS | 0.23 (0.20-0.26, p<0.001) | 0.28 (0.25-0.31, p<0.001) |
| AJCC Clinical T Stage | cT1 | - | - |
|  | cT2 | 1.06 (0.95-1.19, p=0.299) | 1.29 (1.13-1.47, p<0.001) |
|  | cT3 | 1.32 (1.17-1.48, p<0.001) | 1.59 (1.39-1.82, p<0.001) |
|  | cT4 | 2.32 (2.10-2.55, p<0.001) | 2.22 (1.98-2.49, p<0.001) |
| AJCC Clinical N Stage | cN0 | - | - |
|  | cN1 | 0.23 (0.19-0.29, p<0.001) | 0.31 (0.25-0.39, p<0.001) |
|  | cNx | 0.84 (0.77-0.92, p<0.001) | 0.93 (0.84-1.04, p=0.195) |

#

| **Table S3.** Univariable and multivariable Cox regression on long-term overall survival in patients with sarcoma tumors | | | |
| --- | --- | --- | --- |
|  |  | HR (univariable) | HR (multivariable) |
| Center volume | Quintile 1 | - | - |
|  | Quintile 2 | 0.84 (0.77-0.90, p<0.001) | 0.95 (0.87-1.03, p=0.184) |
|  | Quintile 3 | 0.80 (0.74-0.87, p<0.001) | 0.94 (0.85-1.03, p=0.174) |
|  | Quintile 4 | 0.71 (0.66-0.77, p<0.001) | 0.86 (0.77-0.95, p=0.005) |
|  | Quintile 5 | 0.74 (0.68-0.80, p<0.001) | 0.93 (0.83-1.04, p=0.205) |
| Facility Type | Community | - | - |
|  | Integrated | 0.83 (0.77-0.90, p<0.001) | 0.96 (0.88-1.05, p=0.329) |
|  | Academic | 0.81 (0.76-0.86, p<0.001) | 1.02 (0.94-1.11, p=0.682) |
| Facility Location | Northeast | - | - |
|  | Midwest | 0.97 (0.90-1.05, p=0.504) | 0.91 (0.84-0.99, p=0.022) |
|  | South | 1.00 (0.93-1.08, p=0.942) | 0.91 (0.84-0.99, p=0.023) |
|  | West | 0.90 (0.83-0.98, p=0.012) | 0.94 (0.86-1.03, p=0.176) |
| Hospital Distance | <12.5 miles | - | - |
|  | 12.5-49.9 miles | 1.01 (0.95-1.08, p=0.684) | 1.06 (1.00-1.13, p=0.069) |
|  | >/=50 miles | 0.97 (0.90-1.04, p=0.356) | 1.04 (0.95-1.14, p=0.385) |
| Year of Diagnosis | 2004-2005 | - | - |
|  | 2006-2007 | 0.90 (0.82-0.99, p=0.028) | 0.94 (0.85-1.03, p=0.169) |
|  | 2008-2009 | 0.89 (0.82-0.98, p=0.017) | 0.98 (0.89-1.08, p=0.712) |
|  | 2010-2011 | 0.82 (0.75-0.91, p<0.001) | 0.92 (0.83-1.01, p=0.092) |
|  | 2012-2013 | 0.84 (0.76-0.92, p<0.001) | 0.96 (0.87-1.06, p=0.445) |
|  | 2014-2015 | 0.76 (0.67-0.86, p<0.001) | 0.84 (0.74-0.96, p=0.009) |
|  | 2016-2017 | 0.75 (0.67-0.83, p<0.001) | 0.87 (0.78-0.98, p=0.020) |
| Age at diagnosis | <55 | - | - |
|  | 55-59 | 1.08 (0.98-1.19, p=0.123) | 1.12 (1.02-1.24, p=0.022) |
|  | 60-64 | 1.22 (1.12-1.34, p<0.001) | 1.26 (1.15-1.39, p<0.001) |
|  | 65-69 | 1.24 (1.13-1.35, p<0.001) | 1.23 (1.10-1.38, p<0.001) |
|  | 70-74 | 1.71 (1.56-1.87, p<0.001) | 1.59 (1.42-1.78, p<0.001) |
|  | 75-79 | 2.03 (1.85-2.23, p<0.001) | 1.93 (1.72-2.18, p<0.001) |
|  | 80-84 | 2.54 (2.30-2.82, p<0.001) | 2.25 (1.98-2.55, p<0.001) |
|  | 85+ | 3.59 (3.17-4.07, p<0.001) | 3.00 (2.59-3.48, p<0.001) |
| Sex | Male | - | - |
|  | Female | 0.80 (0.75-0.84, p<0.001) | 0.80 (0.75-0.84, p<0.001) |
| Race | White | - | - |
|  | Other | 0.95 (0.88-1.02, p=0.138) | 0.95 (0.88-1.03, p=0.183) |
| CDCC | 0 | - | - |
|  | 1-2 | 1.33 (1.25-1.41, p<0.001) | 1.21 (1.14-1.29, p<0.001) |
|  | 2+ | 2.08 (1.74-2.48, p<0.001) | 1.73 (1.45-2.08, p<0.001) |
| Insurance Status | Medicare | - | - |
|  | Medicaid | 0.88 (0.78-0.98, p=0.021) | 1.18 (1.03-1.35, p=0.014) |
|  | Private | 0.59 (0.55-0.62, p<0.001) | 0.88 (0.80-0.95, p=0.003) |
|  | Not Insured / Other | 0.74 (0.67-0.82, p<0.001) | 0.96 (0.85-1.09, p=0.522) |
| Education level | >21% | - | - |
|  | 13%-20.9% | 1.29 (1.19-1.39, p<0.001) | 1.12 (1.03-1.22, p=0.006) |
|  | 7%-12.9% | 1.18 (1.10-1.28, p<0.001) | 1.15 (1.06-1.25, p=0.001) |
|  | <7% | 1.10 (1.01-1.19, p=0.024) | 1.20 (1.09-1.32, p<0.001) |
| Medical Income | </=$47,999 | - | - |
|  | $48,000-$62,999 | 0.90 (0.84-0.96, p=0.002) | 0.85 (0.79-0.91, p<0.001) |
|  | $63,000 + | 0.72 (0.68-0.77, p<0.001) | 0.75 (0.70-0.82, p<0.001) |
| Residence | Metro | - | - |
|  | Urban | 1.08 (1.00-1.17, p=0.037) | 1.01 (0.93-1.10, p=0.785) |
|  | Rural | 0.77 (0.69-0.86, p<0.001) | 0.81 (0.72-0.92, p=0.001) |
| Tumor Histology | Liposarcoma | - | - |
|  | Leiomyosarcoma | 1.37 (1.29-1.46, p<0.001) | 1.60 (1.49-1.71, p<0.001) |
|  | Other Histology | 2.26 (2.03-2.50, p<0.001) | 2.44 (2.19-2.71, p<0.001) |
|  | Sarcoma NOS | 2.93 (2.73-3.14, p<0.001) | 2.77 (2.57-2.99, p<0.001) |
| AJCC Clinical T Stage | cT1 | - | - |
|  | cT2 | 0.85 (0.78-0.92, p<0.001) | 0.84 (0.76-0.91, p<0.001) |
|  | cT3 | 1.00 (0.92-1.08, p=0.922) | 1.06 (0.98-1.16, p=0.164) |
|  | cT4 | 0.91 (0.85-0.98, p=0.010) | 1.26 (1.17-1.36, p<0.001) |
| AJCC Clinical N Stage | cN0 | - | - |
|  | cN1 | 2.57 (2.29-2.89, p<0.001) | 1.86 (1.65-2.09, p<0.001) |
|  | cNx | 1.20 (1.13-1.27, p<0.001) | 1.13 (1.06-1.21, p<0.001) |
| Allocation to surgery | No | - | - |
|  | Yes | 0.51 (0.48-0.54, p<0.001) | 0.60 (0.57-0.64, p<0.001) |
